# Personality Type D and Psychophysiological Stress Reactivity During Mental Stress in Young Healthy Individuals

**DOI:** 10.3390/bs15070852

**Published:** 2025-06-24

**Authors:** Alexey N. Sumin, Natalia N. Zagorskaya, Anna V. Shcheglova, Anatoly A. Shipilov, Daniil Z. Kostylbaev, Elena A. Shikanova, Ingrid Y. Prokashko

**Affiliations:** 1 Federal State Budgetary Institution “Research Institute for Complex Issues of Cardiovascular Diseases”, Academician L.S. Barbarash Boulevard, 6, 650002 Kemerovo, Russia; n_zagorskaya@mail.ru; 2 Federal State Budgetary Educational Institution of Higher Education “Kemerovo State Medical University” of the Ministry of Health of the Russian Federation, Voroshilov Street, 22A, 650056 Kemerovo, Russia; hipipov@gmail.com (A.A.S.); danya.kostylbaev@gmail.com (D.Z.K.); shikanova.yelena@mail.ru (E.A.S.); proing59@mail.ru (I.Y.P.)

**Keywords:** mental stress, personality type D, stress reactivity, cardiovascular system, psychophysiological parameters

## Abstract

Persons with personality type D are characterized by an “unhealthy lifestyle”, which is manifested by low physical activity, less healthy eating behavior, and failure to comply with doctors’ recommendations. Persons with personality type D have an inadequate response of hemodynamic parameters to psychoemotional stress; the response of other parameters has not been sufficiently studied. The aim of this study was to investigate the association of personality type D with various psychophysiological parameters of the body during mental stress in healthy individuals. Material and Methods: The study involved 79 students of Kemerovo State Medical University aged 18 to 32 years (mean age 20.7 ± 2.4 years). Psychophysiological diagnostics was carried out using the BOSLAB complex; electromyogram, electrocardiogram, body temperature, respiration, galvanic skin response, and photoplethysmogram data were recorded. The stress testing protocol included cognitive tasks and recovery phases. Additionally, the presence of personality type D in students was assessed using the DS-14 questionnaire. The results of stress tests were compared in groups with the presence/absence of type D. Results: The frequency of detection of type D was high (54.4%). When examining the response of psychophysiological parameters, the most pronounced response to stress tests with mental load was noted for heart rate variability and respiratory system parameters. Individuals with type D personality showed more pronounced sympathetic activation in response to mental stress and a slower recovery at rest. Among the studied parameters, association with personality type D was noted for the following indicators during the mental arithmetic test: heart rate (*p* = 0.022), the Baevsky strain index (*p* = 0.004), respiratory rate (*p* = 0.020), and an indicator of regulatory process adequacy (*p* < 0.001). Conclusion: In the present study, we found differences in the reaction of psychophysiological parameters to mental stress in healthy individuals depending on the presence or absence of personality type D. These data can be useful for developing stress resistance programs and biofeedback training. The possibility of using the above psychophysiological parameters in biofeedback training programs for individuals with personality type D requires further research.

## 1. Introduction

Among the psychosocial factors influencing health behavior in recent years, personality type D (distressful) stands out. Individuals with this personality type are characterized by negative excitability (NA) in response to the challenges of everyday life and the inability to reduce it in effective social interactions (social inhibition = SI) (Denollet, 2005). A strong relationship has previously been shown between Type D personality traits and two Big Five factors, neuroticism and introversion. Both negative affectivity and neuroticism are characterized by high susceptibility to stress and high feelings of fear and stress. Social inhibition is similar to introversion (De Fruyt & Denollet, 2002). Individuals with type D are more prone to unhealthy habits (low physical activity (Wiencierz & Williams, 2017; Yetişir et al., 2025)), have less healthy eating behavior (Booth & Williams, 2015), are less likely to follow doctor’s recommendations, and have lower adherence to treatment (Crawshaw et al., 2016; Y. H. Lin et al., 2020). All these manifestations fit well into the concept of “unhealthy lifestyle” (Buczkowska et al., 2022). As a result, such a lifestyle ultimately contributes to the development of cardiovascular pathology (Kupper & Denollet, 2018). Therefore, personality type D is recognized as one of the psychosocial risk factors for cardiovascular diseases (Belialov et al., 2024). In addition, personality type D is associated with a decrease in the quality of life in almost all studied cohorts of patients (S. R. Kim et al., 2021; Sánchez-Díaz et al., 2023). The latter fact is important in the concept of modern medicine, which proclaims a patient-oriented approach. This approach consists, among other things, in achieving a treatment result that most closely matches the patient’s expectations, which improves their quality of life. It is clear that with this approach, it is necessary to take into account the personal characteristics of the patient (Wu et al., 2025). Among other things, personality type D has an unfavorable prognostic value in patients with cardiovascular diseases. This has been convincingly demonstrated for patients with coronary heart disease, both in individual studies (Sumin & Shcheglova, 2023; Wang et al., 2025) and in a recently published meta-analysis (Lodder et al., 2023).

All these data make it necessary to consider the identification of individuals with personality type D and the development of their resilience and healthy coping mechanisms as an important component of individual preventive measures aimed at improving health behavior (Roohafza et al., 2025; Albus, 2025). The focus of such preventive measures depends on the pathophysiological mechanisms by which personality type D is associated with the development and progression of diseases. The pathophysiological influence of personality type D on clinical indicators and the prognosis of patients is caused not only indirectly through the indirect influence of an unhealthy lifestyle. It is believed that individuals with personality type D also have an inadequate response to the stressful influences of everyday life, characterized by an increased neurohormonal response, which is manifested by an increase in cortisol levels, sympathetic activation, and endothelial dysfunction. Therefore, one of the ways to improve health behavior, quality of life, and prognosis in patients with personality type D is to correct inadequate reactions to stressful influences (Yamaguchi et al., 2025). However, modeling the stress response in laboratory conditions for personality type D has led to conflicting results. Although some patients have increased stress reactivity (O’Riordan et al., 2019; O’Riordan et al., 2020), a number of studies have noted a reduced cardiovascular response to stress for personality type D (Kupper et al., 2013a; O’Riordan et al., 2023a). The reasons for such conflicting results remain unclear; possible reasons include differences in the stressors used in the laboratories (O’Riordan et al., 2020) and the contingents of individuals examined (O’Riordan et al., 2019). Another reason may be the insufficient set of studied psychophysiological parameters during such stressful influences (as a rule, they are limited to blood pressure, heart rate, and heart rate variability (Li et al., 2018; O’Riordan et al., 2023b). Apparently, the problem of stress reactivity in individuals with personality type D has not been sufficiently studied and requires further research. One of the promising methods for assessing the body’s resistance to stress is the use of biofeedback devices with the measurement of certain physiological variables (electromyography, pulse, blood volume, respiratory rate, peripheral temperature, and skin conductivity) during psychophysiological tests (Diaz-Ramos et al., 2021; Figueroa et al., 2023). However, the relations of personality type D with the response of these parameters to stress have not yet been studied. This served as the basis for conducting the present study, the purpose of which was to study the association of personality type D with various psychophysiological parameters of the body during mental stress in healthy individuals.

## 2. Materials and Methods

### 2.1. Study Design, Setting, and Participants

A cross-sectional study was conducted in 79 second-year students of the Kemerovo State Medical University in December 2024. The cohort of students was heterogeneous in terms of ethnicity, age, and gender. The subjects ranged in age from 18 to 32 years (mean age 20.7 ± 2.4 years); there were 31 men and 48 women among the subjects. The subjects belonged to different ethnic groups (Russians—38; Indians—18; representatives of other nationalities—23). The studies were conducted in laboratory conditions in the morning hours with a stable positive state of health and performance. The study was conducted in accordance with the Declaration of Helsinki. The study protocol was approved (date of approval: 15 October 2024) by the Local Ethical Committee of the Research Institute for Complex Issues of Cardiovascular Diseases (Protocol No. 20241015). All study participants signed an informed consent form.

### 2.2. Variables

All participants completed the DS-14 psychological questionnaire to identify personality type D and the Hospital Anxiety and Depression Scale (HADS); they also underwent tests with mental stress load with an assessment of psychophysiological parameters, and an assessment of arterial stiffness was carried out. Data processing complied with current regulations guaranteeing anonymity and the security of information in general. Since the subjects were university students, they were recruited by teachers with whom they had no academic interaction; participation in the study was voluntary and did not have any incentives.

### 2.3. Data Sources/Measurement

#### 2.3.1. Assessment of Personality Type D

All study participants completed either the Russian version of the DS-14 questionnaire (Pushkarev et al., 2019) or, in the case of Indian students, the English version of the questionnaire (Denollet, 2005). For the Russian version of the questionnaire, Cronbach’s alpha was 0.78 for NA and 0.74 for SI. The questionnaire contains 14 questions, which are divided into 2 scales: negative affectivity (NA) of emotions and the degree of social inhibition (SI). Answers to the questions range from “false” (0 points) to “absolutely true” (4 points), including intermediate answers “rather false” (1 point), “difficult to say” (2 points), and “probably true” (3 points). If the subject scores 10 or more points on each scale, then personality type D is determined. For further analysis, groups with and without personality type D were identified.

#### 2.3.2. Hospital Anxiety and Depression Scale (HADS) 

We used the Hospital Anxiety and Depression Scale (HADS) to assess the level of depression and anxiety. We used a version of the questionnaire that was translated and adapted for the Russian language. This questionnaire includes 14 statements, which consist of 2 subscales: “anxiety” (A) and “depression” (D). For each statement, 4 answer options are offered, which reflect the severity of symptom manifestations. Responses are coded depending on the increase in symptom severity (from 0 points to 4 points—with maximum severity). Accordingly, higher scores on the subscales reflect more pronounced manifestations of anxiety or depression. High Cronbach’s alpha values for all questions in the Russian version of the HADS (α = 0.90), as well as for the anxiety (α = 0.86) and depression (α = 0.84) subscales, indicate good internal consistency of the questionnaire.

#### 2.3.3. Psychophysiological Testing

For the study, the multichannel biofeedback complex “BOSLAB” professional Plus BI-012-2 (OOO “Computer Biocontrol Systems”, Novosibirsk, Russian Federation) was used. The stress testing protocol consisted of sessions that allowed assessing the characteristics of the body’s stress reactivity and subsequent recovery ability. In total, psychophysiological parameters were assessed during five sessions: a one-minute rest session (during which the initial physiological parameters were recorded), then—as a mild stressor—a cognitive task on oral arithmetic counting for three minutes, then another minute of rest, and then a second cognitive task, the “Stroop test” (Stroop, 1992), for three minutes, and a final rest session. During each session, physiological data were recorded, and parameters derived from them were calculated. The following physiological parameters of the subjects were recorded using the biofeedback complex: heart rate, depth and duration of breathing, photoplethysmography, electromyography, and galvanic skin response. The registration of electrocardiography (ECG) in the II modified lead and the depth and duration of breathing was carried out using a sensor secured with an induction belt in the sternum area. Photoplethysmography (PPG) was recorded on the distal phalanx of the first finger of the hand, and electromyography (EMG) was recorded from the frontal muscles. The temperature of the peripheral skin was assessed using a sensor placed on the distal phalanx of the second finger of the hand. The galvanic skin response (GSR) was assessed using sensors attached to the proximal phalanges of the second and third fingers of the hand on the palmar side.

Using the “raw” signals obtained during the instrumental study on the biofeedback complex, a number of physiological parameters and indices were calculated. Among them were R-R intervals, heart rate (HR), and respiratory sinus arrhythmia (RSA). The state of the vascular bed was characterized by the following parameters: pulse wave propagation time (PWPT) and PPG systolic wave amplitude. PWPT characterizes the elasticity of the main vessels, and PPG amplitude reflects the volumetric blood flow at the registration site and the state of the arteries of the microcirculatory bed. The frequency of respiratory movements, the frequency of the mode of respiratory movements; the duration of the respiratory cycle (RCD), the ratio of the duration of inspiration to the duration of expiration (Rio), and the number of R-R intervals in one respiratory cycle (NNbc) characterized the state of the respiratory system. Additionally, the integral index of tension from the frontal muscles (iEMG), skin conductivity, and skin temperature on the Fahrenheit scale were measured.

In addition, a number of indicators related to the group of heart rate variability indices were assessed. The ratio of low- and high-frequency waves of the heart rate (LF/HF) allows us to assess the ratio of sympathetic or parasympathetic influences on the heart rate. The Baevsky strain index (SI) reflects the predominance of central or peripheral mechanisms in the regulation of the heart rate and is an integral marker of the level of stress and the body’s adaptation to it. The Baevsky indicator of the adequacy of regulatory processes (IAPR) shows the degree of sympathetic influences on the heart rate in relation to the leading function of the sinus node. This group of indices was of particular interest due to the potential significance of use in the early diagnosis of structural and functional abnormalities in the functioning of the cardiovascular system, as well as in assessing the general condition of the body.

#### 2.3.4. Assessment of Vascular Stiffness Indices

Vascular stiffness indices were assessed using a VaSera VS-1000 sphygmomanometer (Fukuda, Japan). The following indices were measured at rest: heart rate, cardio-ankle vascular index (CAVI), and ankle–brachial index (ABI).

### 2.4. Bias/Study Size

To roughly calculate the sample size, we used the a priori sample size calculator for Student’s *t*-tests. With an anticipated effect size (Cohen’s d) of 0.6, a probability level of 0.05, and a minimum total sample size of 72, the desired statistical power level was 0.8. Since our study sample was 79 people, the desired statistical power was achieved.

### 2.5. Statistical Methods

Statistical processing of the results was performed using the application packages “Statistica 10.0 for Windows” (StatSoft Inc., Tulsa, OK, USA) and SPSS 17.0 (IBM, Armonk, New York, NY, USA). The distribution of quantitative variables was tested for normality using the Kolmogorov–Smirnov test. Since the distribution of all quantitative characteristics differed from normal, they are presented as the median (Me) and the lower (LQ) and upper (UQ) quartiles. Groups were compared using the Mann–Whitney criterion. To compare the dynamics of the physiological parameters of the participants throughout the study, the Friedman ANOVA criterion was used.

To assess the association of type D with the dynamics of psychophysiological indicators during mental load tests, multiple logistic regression analysis was performed (Forward Stepwise LR method). The mental test indicators for which there were reliable differences between the groups were included as independent variables in the models. To identify a possible relationship between the indicators in mental tests (indicator of regulatory process adequacy, respiratory rate, heart rate, and Baevsky’s strain index) and the components of personality type D (type D, ZscoreNA, ZscoreSI, and zNA × zSI), a linear regression analysis was additionally conducted. The critical level of significance (p) was taken to be 0.05.

## 3. Results

### 3.1. Participants and Descriptive Data

#### General Characteristics of Medical Students in Groups With/Without Type D Personality

In the cohort of healthy students we examined, a high frequency of individuals with type D personality was noted (43 people or 54.4%). When comparing the groups with/without type D personality, no differences were found between them in age, gender, and ethnic composition (Table 1). The scores on the subscales of the DS-14 questionnaire (NA and SI) were naturally higher among students with type D personality. Also, expectedly higher levels of anxiety and depression were found in the group with type D compared to the group without type D (10 points versus 7 points, *p* = 0.003 for anxiety; 9.0 versus 6.0, *p* = 0.015 for depression).

The study on the VaSera VS-1000 sphygmomanometer did not reveal any significant differences in heart rate and vascular indices reflecting the state of the vascular wall (ABI and SLSI) among individuals with type D personality compared to other students (Table 1).

### 3.2. Main Results

#### 3.2.1. Dynamics of Cardiovascular System Functioning Indicators During Mental Stress Tests in Groups With/Without Type D Personality

Table 2 presents the indicators based on heart rate variability and the state of the vascular wall. In general, in both groups of subjects, significant dynamics of three indicators were noted against the background of mental stress—an increase in heart rate, shortening of R-R intervals, and a decrease in SI, which reflects the state of sympathetic activation. When comparing the groups against the background of mental stress, higher values of IAPR were noted in the group with type D (*p* = 0.026 for the mental arithmetic test and *p* = 0.031 for the Stroop test). This means that the influence of sympathetic regulatory effects on the heart rate in this group of subjects increased under stress. Differences between the groups in the recovery phase after the tests (rest session 3) were also revealed. In the group with type D, lower values of RSA (*p* = 0.024) and higher SI (*p* = 0.011) were noted, along with a tendency towards higher values of IAPR (*p* = 0.059). Apparently, these data reflect the fact that the processes of recovery after stress loads proceed differently in individuals with/without type D. For other studied parameters (LF/HF ratio and PTT amplitude), no significant dynamics were revealed during the mental test, nor were differences revealed between the groups throughout all sessions.

#### 3.2.2. Dynamics of Respiratory System Functioning Indicators During Mental Stress Tests in Groups With/Without Type D Personality

The students’ respiratory system functioning indicators also changed significantly during the stress testing; however, no strict pattern was observed among these changes (Table 3). Thus, the respiratory rate as a whole was slightly lower by the end of testing compared to the initial value for both type D and non-type D personality representatives (*p* = 0.05 and *p* = 0.01, respectively). This was accompanied by an increase in the breathing mode frequency (*p* = 0.001 and *p* = 0.002, respectively) and a decrease in the breathing mode frequency by the end of testing for type D personality (*p* = 0.032). At the same time, during the mental arithmetic test, the respiratory rate was significantly lower for students with type D personality (*p* = 0.035). The ratio Rio changed differently during the sessions for type D and non-D personality types: by the end of testing, it decreased for type D and increased in the absence of type D (*p <* 0.01 and *p* = 0.041), while during the Stroop test, this value decreased (students pronounced the names of colors written on the monitor out loud while exhaling).

#### 3.2.3. Dynamics of Other Functional Indicators During Mental Stress Tests in Groups With/Without Type D Personality

Among the other parameters studied (Table 4), an increase in the temperature of the peripheral skin was noted by the end of the study in all students, both with type D and without it (*p <* 0.001 and *p* = 0.002, respectively). PPG amplitude by the end of testing increased in students with type D and decreased in the group without type D (*p <* 0.001 in both cases, Table 4).

According to the integrated EMG data, the tension of the frontal muscles (as a probable marker of the stress response) during testing in representatives of personality type D increased from the initial level by the final session (*p* = 0.019), while no such reaction was observed in the other students (*p* = 0.539).

### 3.3. Other Analyses

#### 3.3.1. Psychophysiological Indices of Mental Stress Associated with Personality Type D

To identify psychophysiological parameters in mental stress associated with personality type D, we used binary logistic regression. The model included all studied indicators presented in Table 2, Table 3 and Table 4 (namely, RR, HR, PWPT, LF/HF, SI, IARP, RSA, RRate, BMF, RCD, RIO, NNbc, PPG, iEMG, SC, Temp) in stress sessions (arithmetic calculation and Stroop test), as well as age, gender, and ethnicity. The results of this analysis are presented in Table 5. An independent significant association with type D personality (χ^2^(4) = 25.806, *p* < 0.001, Suppl. Table S1) was observed only for the following mental arithmetic test scores: heart rate (B = −0.084, *p* = 0.022), Baevsky SI (B = −0.032, *p* = 0.004), respiratory rate (B = −0.203, *p* = 0.020), and IARP (B = 0.151, *p* < 0.001). This model explained 38.5% (Nagelkerke R^2^) of the variance in type D and correctly classified 75.0% of cases (Suppl. Tables S2 and S3).

#### 3.3.2. Association of Indicators During Mental Arithmetic Test with Components of Personality Type D

In the previous sections of the article, we analyzed personality type D as a dichotomous indicator. In contrast, in this section, we attempt to compare the association of various components of the type D concept (type D as a dichotomous indicator, with values for the type D subscales—NA and SI—as continuous data, as well as the synergy indicator of these indicators) with psychophysiological indicators during mental stress. As the latter, we used indicators that had an independent association with type D in binary logistic regression (Table 6). When assessing the multicollinearity of multiple linear regression models, the values of the VIF indicator were less than 5 in all cases. However, the results of multiple linear regression analysis revealed rather modest associations—only the Zscore SI indicator had a significant association with the indicator of regulatory process adequacy (*p* = 0.013) and Baevsky’s strain index (*p* = 0.038) in the mental arithmetic test. Perhaps, this analysis is simply not suitable for identifying differences between the components of the personality type D construct.

## 4. Discussion

Until now, no comprehensive studies of several physiological parameters in response to stress using biofeedback devices have been conducted in individuals with personality type D. In the present study, we found differences in the reaction of psychophysiological parameters to mental stress in healthy individuals depending on the presence or absence of personality type D. In type D, higher PAPR values were observed in response to stress factors (mental arithmetic test, Stroop test), which can be regarded as increased sympathetic activation. The differences observed in the groups after the tests (lower RSA values, higher Baevsky SI, and increased PPG amplitude and frontal muscle tension in type D) can be interpreted as a delayed recovery in the group with personality type D.

Previous studies on the effects of stress on individuals with personality type D focused on the stress reactivity of hemodynamic parameters. At the same time, the initial assumption about the increased hemodynamic reaction in response to stress in type D (Williams et al., 2009; Kupper et al., 2013b) was not subsequently confirmed (O’Riordan et al., 2023a; Kelly-Hughes et al., 2014). It was suggested that the stress of everyday life has a longer negative impact on hemodynamics in type D, which ultimately results in its unfavorable prognostic effect (Li et al., 2018; Molloy et al., 2008). However, recent studies, on the contrary, have revealed less pronounced hemodynamic reactions in response to moderate mental stress in personality type D. Thus, women with personality type D had lower SBP reactivity in the mental arithmetic test (O’Riordan et al., 2023b). Also, patients with chronic heart failure with personality type D demonstrated an inadequate response to acute social stress, characterized by a blunted heart rate response (Kupper et al., 2013a).

Several explanations have been proposed for these unexpected results that do not fit into the original concept. First, such tests in laboratory conditions during individual examination do not cause sufficient motivation in individuals with personality type D to perform them thoroughly, which is accompanied by a weaker hemodynamic response. Second, more stressful situations for individuals with type D are situations that are more consistent with real-life situations (e.g., public speaking). In such cases, they exhibit increased hemodynamic stress reactivity (O’Riordan et al., 2019). On the other hand, reactions to stress from the body are not limited to hemodynamic parameters. It is known that individuals with type D have an increased neurohormonal response to stress. It has been shown that personality type D was associated with long-term dysfunction of the hypothalamic–pituitary–adrenal axis in survivors of acute cardiac events. In patients with acute coronary events, daily cortisol secretion was higher in type D than in patients without type D (Molloy et al., 2008). In type D personality, endothelial dysfunction is detected in patients with coronary heart disease (Denollet et al., 2018), and against the background of mental stress, a more pronounced decrease in the vasodilator response in the reactive hyperemia test is noted (Sumin et al., 2011).

In individuals with high levels of psychological distress, a delayed recovery from the stress of public speaking was noted due to persistent sympathetic activity and reduced cardiovagal modulation due to decreased baroreflex sensitivity (Koutnik et al., 2014). Accordingly, dysautonomia is assumed to be a potential factor linking high levels of distress and the development of cardiovascular diseases. The data of the present study are quite consistent with these results.

Stress reactivity of other psychophysiological indicators in type D personality has been poorly studied. To date, these parameters have been studied in other cohorts of healthy individuals and patients. Thus, the assessment of skin conductivity during the mental arithmetic test has been proposed for future diagnosis and prognosis of depression based on an objective interpretation of depressive states (A. Y. Kim et al., 2019). In individuals with psychosomatic disorders against the background of stress (playing cyberball), an increase in skin conductivity was noted, indicating stimulation of the sympathetic nervous system at this time (Thurner et al., 2022). A recent study proposed an individualized approach to assessing psychoemotional stress using the skin conductivity index in real time during continuous performance of a task of varying difficulty levels (Kriklenko et al., 2024).

The use of electromyography during stress tasks also allows us to assess various aspects of maladaptive stress responses. For example, when studying facial muscle activity using EMG, it is possible to identify differences in self-esteem associated with stress (the activity of either the corrugator muscles—frowning—or the zygomatic muscles—smiling) (Kroll et al., 2021). Also, recording muscle tension using surface electromyography in response to the “Stroop test” was used to assess the effect of breathing exercises on stress reactivity (Liang et al., 2023). Finally, EMG-based biofeedback training has proven its effectiveness in a complex of rehabilitation measures due to the improvement of both the physical and psychological state in the presence of a combination of somatic pathology with symptoms of anxiety, depression, and sleep disorders (Sadora et al., 2023). 

Meanwhile, the assessment of several physiological parameters at once during stress is a promising method for assessing stress reactivity. Therefore, a new index was developed to measure human resilience to stress based on changes in certain parameters (electromyography, which is a muscle response, pulse, respiratory rate, peripheral temperature, and skin conductivity) using a biofeedback device during a 10-minute psychophysiological stress test. To calculate the index, the authors used unsupervised machine learning methods to calculate intercluster distances. Calculating such an index for an individual can have a wide range of applications, including understanding and supporting mental health. Accordingly, patients can track their resilience to stress, which ultimately contributes to improving not only their daily lives, but also disease prevention (Diaz-Ramos et al., 2021). One of the first experiences with a multicomponent program including biofeedback training showed that it contributed to positive changes in the stress resilience index (Diaz-Ramos et al., 2021) and in managing psychophysiological reactions to academic stress (Figueroa et al., 2023). 

These publications demonstrate the promise of our approach with the assessment of a large number of physiological parameters during mental stress tests, which will contribute to the development of further programs for both assessing stress reactivity and increasing stress resistance in individuals with personality type D. 

What is the significance of this study for further research in this area? It has recently been shown that in individuals with stable coronary artery disease, blunted cardiovascular reactivity to mental stress was associated with adverse outcomes (Moazzami et al., 2023). Personality type D is also associated with inadequate responses to mental stress and a negative impact on both the quality of life and the prognosis of patients. Therefore, it is important to search for optimal ways to adapt individuals with personality type D to the stressful influences of everyday life, as well as to reduce adverse reactions to stress in such individuals (Cao et al., 2016; Lee et al., 2018). One such method may be the use of biofeedback devices to increase stress resistance in individuals with personality type D. The devices used for biofeedback training allow it to be carried out using various sensors. It is quite possible that the most suitable parameters for biofeedback training will be those with the most pronounced reaction to stress, and for individuals with type D, those with the most pronounced differences in reaction compared to individuals without type D. The data of the present study allow us to propose such variants of biofeedback training. 

It should be recognized that additional studies are needed to confirm and expand on the current results. Firstly, similar studies should be conducted on other categories of healthy individuals (middle-aged individuals, older individuals, individuals with different educational levels, etc.). Secondly, it is necessary to evaluate the influence of type D on the dynamics of psychophysiological parameters in patients, primarily with cardiovascular diseases (taking into account the above data on the prognostic value of type D in such individuals). Thirdly, it is necessary to evaluate various variants of biofeedback training (temperature–myographic training, EEG training, respiratory rate, heart rate, etc.) in individuals of all the above categories in order to select the most suitable training variants for individuals with type D. And finally, it will then be necessary to evaluate the effectiveness of the course application of the developed biofeedback training programs in individuals with type D.

### Limitations of the Study

When analyzing the results of this study, it is necessary to take into account its limitations. First, the number of participants examined was relatively small, which may affect the statistical significance of the results obtained. Second, we examined young healthy individuals, so these data should be extrapolated with caution to older individuals and those with cardiovascular diseases. Third, the studied cohort of individuals (medical students) differed in their level of education and medical awareness, so our data should be used with caution for the general population. In addition, we studied personality type D as a dichotomous variable (this served as the basis for dividing into groups and was used in binary logistic regression). As shown in a number of publications (Lodder et al., 2021), such an examination of the personality type D construct has limitations and may overestimate the prognostic value of type D. However, when attempting to consider the various components of type D in the analysis of multiple linear regression, we were unable to identify significant associations with psychophysiological parameters under mental stress. Apparently, in the cohort we studied, considering personality type D as a dichotomous parameter allowed us to identify the above associations. Furthermore, a recent study showed that type D personality was significantly associated with MACE in patients with stable coronary artery disease, regardless of whether it was defined dichotomously or continuously (T. K. Lin et al., 2025). An additional limitation is the use of the scale in two languages, which may hinder the comparability of their results.

## 5. Conclusions

In the cohort of healthy individuals we studied, a high frequency of personality type D was observed (54.4%). When examining the response of psychophysiological parameters, the most pronounced response to stress tests with mental load was noted for heart rate variability and respiratory system parameters. Individuals with type D personality showed more pronounced sympathetic activation in response to mental stress and a slower recovery at rest. Among the studied parameters, association with personality type D was noted for the following indicators during the mental arithmetic test: heart rate (*p* = 0.022), Baevsky SI (*p* = 0.004), respiratory rate (*p* = 0.020), and IARP (*p* <0.001). The possibility of using the above psychophysiological parameters in biofeedback training programs for individuals with personality type D requires further research.

## Figures and Tables

**Table 1:** General characteristics of medical students in groups with/without type D personality.

Indicators (Me (LQ; UQ)) or n (%)	Non-Type D (n = 36)	Type D (n = 43)	*p*
Age, years	20.8 [19.0; 22.0]	20.8 [19.0; 22.0]	0.640
Men	16 (44.4%)	15 (34.9%)	0.389
Russians	19 (53.8%)	19 (44.2%)	0.717
Indians	7 (19.4%)	11 (25.6%)	
Other ethnicities	10 (27.8%)	13 (30.2%)	
DS-14			
Negative affectivity, points	6.6 [3.0; 9.0]	15.3 [12.0; 18.0]	<0.001
Social inhibition, points	9.28 [7.0; 11.5]	14.8 [12.0; 16.0]	<0.001
HADS			
Anxiety, points	7.0 [5.0; 9.0]	10.0 [7.0; 11.0]	0.003
Depression, points	6.0 [4.0; 10.0]	9.0 [7.0; 11.0]	0.015
VaSera VS-1000			
Heart rate, beats/min	77 [68; 87]	71 [64.5; 80.5]	0.123
Right CAVI	5.5 [4.8; 6.2]	5.8 [4.9; 6.0]	0.443
Left CAVI	5.4 [4.9; 6.4]	5.9 [4.9; 6.2]	0.355
Right ABI	1.02 [0.92; 1.10]	0.99 [0.93; 1.10]	0.806
Left ABI	1.01 [0.94; 1.08]	1.05 [0.93; 1.16]	0.341

Note: ME (LQ; UQ)—median with upper and lower quartiles; CAVI—cardio-ankle vascular index right; ABI—ankle–brachial index right.

**Table 2:** Dynamics of cardiovascular system functioning indicators during mental stress tests in groups with/without type D personality.

	Рersonality Type	Rest Session #1	Мental Arithmetic Test	Rest Session #2	Stroop Test	Rest Session #3	*р*
R-R intervals, ms	Type D	712.62 [646.29; 795.45]	655.64 [646.30; 733.26]	730.08 [672.24; 783.17]	654.01 [590.98; 728.53]	721.35 [682.51; 836.35]	<0.001
	Type not D	725.20 [686.74; 793.53]	679.63 [609.52; 706.14]	719.99 [669.30; 774.67]	650.61 [610.07; 692.53]	743.01 [676.13; 797.53]	<0.001
	*р*	0.384	0.519	0.794	0.910	0.809	
HR, beats/min	Type D	85.0 [75.8; 93.2]	91.6 [82.2; 99.7]	83.1 [76.7; 89.7]	93.4 [82.4; 101.8]	83.7 [72.2; 88.1]	<0.001
	Type not D	83.5 [75.9; 88.0]	88.4 [85.1; 98.6]	83.9 [78.3; 90.3]	92.7 [86.8; 100.5]	81.0 [75.5; 89.2]	<0.001
	*р*	0.357	0.513	0.764	0.910	0.817	
LF/HF	Type D	1.09 [0.45; 2.14]	1.54 [0.79; 3.67]	1.75 [0.77; 2.53]	1.18 [0.78; 2.39]	1.00 [0.54; 2.29]	0.282
	Type not D	1.33 [0.61; 2.39]	1.78 [1.01; 2.53]	1.16 [0.63; 2.29]	1.78 [1.01; 2.53]	1.27 [0.58; 2.28]	0.102
	*р*	0.669	0.749	0.251	0.410	0.988	
RSA, ms	Type D	79.60 [53.54; 121.37]	110.95 [71.99; 139.12]	82.90 [64.03; 116.63]	89.24 [65.78; 153.06]	79.07 [62.98; 117.27]	0.311
	Type not D	92.22 [72.42; 138.27]	96.98 [76.85; 135.89]	111.33 [73.52; 143.75]	96.69 [80.55; 143.91]	105.42 [89.15; 163.25]	0.132
	*р*	0.301	0.690	0.159	0.486	0.024	
SI	Type D	102.04 [62.64; 189.16]	62.82 [42.71; 100.61]	88.83 [53.08; 125.71]	79.67 [45.33; 140.71]	105.13 [60.47; 131.46]	<0.001
	Type not D	93.03 [53.89; 150.77]	59.95 [34.58; 102.12]	59.72 [41.30; 122.50]	69.37 [45.38; 91.59]	59.07 [34.62; 116.37]	0.012
	*р*	0.475	0.605	0.116	0.247	0.011	
IARP	Type D	57.90 [42.93; 69.28]	56.90 [45.59; 69.54]	53.14 [42.46; 65.60]	57.82 [44.66; 70.76]	52.94 [42.74; 66.97]	0.427
	Type not D	50.39 [41.96; 58.42]	45.73 [35.33; 59.09]	48.62 [33.45; 61.92]	49.50 [39.91; 59.38]	43.70 [32.77; 63.92]	0.196
	*р*	0.226	0.026	0.189	0.031	0.059	
PWPT, ms	Type D	182.62 [141.03; 224.44]	177.28 [142.13; 237.15]	169.42 [142.81; 247.51]	180.48 [150.25; 228.12]	195.71 [141.65; 345.06]	0.985
	Type not D	203.00 [141.37; 277.73]	185.99 [147.92; 227.67]	189.09 [145.21; 342.52]	199.24 [145.20; 281.19]	237.10 [141.63; 413.28]	0.176
	*р*	0.703	0.663	0.291	0.338	0.417	

Note: R-R intervals—R-R interval duration; HR—heart rate; RSA—respiratory sinus arrhythmia; LF/HF—low- to high-frequency ratio; SI—Baevsky’s strain index; IARP—indicator of the adequacy of regulatory processes; PWPT—pulse wave propagation time.

**Table 3:** Dynamics of respiratory system functioning indicators during mental stress tests in groups with/without type D personality.

	Рersonality Type	Rest Session #1	Мental Arithmetic Test	Rest Session #2	Stroop Test	Rest Session #3	*р*
Resp. rate, breaths/min	Type D	18.7 [15.6; 20.8]	17.9 [14.5; 20.3]	17.9 [14.1; 20.4]	16.1 [14.2; 18.3]	17.2 [14.3; 20.4]	0.050
	Type not D	16.9 [14.6; 18.3]	19.6 [16.6; 22.0]	16.4 [14.8; 19.3]	16.8 [15.2; 19.6]	15.3 [13.0; 19.4]	0.010
	*р*	0.221	0.035	0.496	0.198	0.238	
BMF, Hz	Type D	0.267 [0.217; 0.317]	0.250 [0.150; 0.300]	0.267 [0.150; 0.317]	0.183 [0.133; 0.217]	0.258 [0.175; 0.342]	0.032
	Type not D	0.267 [0.217; 0.317]	0.258 [0.192; 0.333]	0.242 [0.200; 0.308]	0.200 [0.167; 0.233]	0.225 [0.117; 0.300]	0.181
	*р*	0.972	0.151	0.880	0.255	0.250	
RCD, sec.	Type D	3.443 [3.197; 4.060]	4.072 [3.384; 4.938]	4.175 [3.263; 5.366]	4.517 [3.954; 5.046]	4.084 [3.249; 5.016]	0.001
	Type not D	3.696 [3.343; 4.767]	3.514 [3.124; 4.499]	4.148 [3.444; 5.080]	4.100 [3.682; 5.013]	4.723 [3.602; 5.705]	0.002
	*р*	0.306	0.059	0.704	0.201	0.316	
Rio	Type D	0.759 [0.644; 0.869]	0.778 [0.513; 1.129]	0.726 [0.633; 1.032]	0.562 [0.429; 0.710]	0.699 [0.593; 1.037]	<0.001
	Type not D	0.667 [0.607; 0.805]	0.649 [0.552; 1.056]	0.713 [0.647; 0.921]	0.595 [0.467; 0.898]	0.819 [0.632; 1.082]	0.041
	*р*	0.123	0.633	0.775	0.375	0.272	
NNbc	Type D	4.632 [4.222; 5.706]	6.096 [5.167; 7.811]	5.462 [4.642; 7.250]	6.549 [5.559; 7.571]	5.258 [4.257; 6.967]	<0.001
	Type not D	4.765 [3.850; 6.231]	5.596 [4.679; 6.850]	5.333 [4.420; 7.000]	6.037 [5.145; 7.621]	5.828 [4.478; 7.283]	<0.001
	*р*	0.977	1,122	0.954	0.290	0.581	

Note: Resp. rate—respiratory rate; BMF—breathing mode frequency; RCD—respiratory cycle duration; Rio—ratio of inhalation time to exhalation time; NNbc—number of R-R intervals in one respiratory cycle.

**Table 4:** Dynamics of other functional indicators during mental stress tests in groups with/without type D personality.

	Рersonality Type	Rest Session #1	Мental Arithmetic Test	Rest Session #2	Stroop Test	Rest Session #3	*р*
PPG amplitude	Type D	161.2 [99.1; 212.1]	163.8 [101.9; 280.2]	185.4 [104.2; 288.2]	132.5 [97.1; 189.4]	173.1 [103.7; 312.4]	<0.001
	Type not D	136.9 [104.3;226.9]	130.7 [105.5; 178.2]	180.9 [146.1; 266.6]	131.0 [110.4; 161.4]	130.4 [109.5; 233.2]	<0.001
	*р*	0.879	0.332	0.571	0.840	0.482	
iEMG, mcV	Type D	12.7 [10.3; 18.3]	14.5 [10.6; 21.1]	12.6 [8.3; 17.8]	14.3 [10.6; 19.1]	13.6 [9.1; 18.5]	0.019
	Type not D	11.5 [8.6; 17.0]	11.9 [9.6; 16.6]	12.5 [8.5; 15.1]	12.0 [9.3; 15.3]	12.5 [8.0; 18.4]	0.539
	*р*	0.332	0.82	0.647	0.071	0.712	
SC, mcS	Type D	6.0 [2.9; 8.7]	8.1 [5.3; 11.6]	8.2 [5.3; 12.4]	9.3 [5.5; 14.4]	9.8 [6.0; 11.8]	<0.001
	Type not D	6.6 [4.2; 11.1]	8.5 [4.8; 13.8]	8.4 [5.1; 14.7]	9.3 [5.7; 16.2]	9.3 [6.0; 15.3]	<0.001
	*р*	0.451	0.598	0.654	0.654	0.612	
Temp, F	Type D	79.4 [76.1; 89.7]	79.4 [75.8; 89.5]	81.2 [75.4; 91.5]	80.7 [75.1; 91.1]	80.3 [74.6; 90.0]	<0.001
	Type not D	79.0 [75.6; 88.6]	78.8 [75.0; 88.4]	79.9 [74.3; 88.0]	80.8 [74.6; 89.2]	80.3 [74.4; 88.3]	0.002
	*р*	0.506	0.500	0.532	0.794	0.802	

Note: PPG amplitude—systolic wave amplitude during photoplethysmography; iEMG—integrated electromyography; SC—skin conductance; Temp—temperature.

**Table 5:** Psychophysiological indices of mental stress associated with personality type D (binary logistic regression analysis, forward likelihood ratio).

		B	S.E.	Wald	df	Sig.	Exp(B)
Step 1	IARP MAT	0.032	0.014	5.031	1	0.025	1.032
	Constant	−1.508	0.773	3.801	1	0.051	0.221
Step 2	Respiratory Rate MAT	−0.145	0.064	5.106	1	0.024	0.865
	IARP MAT	0.034	0.014	5.497	1	0.019	1.034
	Constant	0.995	1.329	0.561	1	0.454	2.705
Step 3	SI MAT	−0.027	0.010	6.626	1	0.010	0.974
	Respiratory Rate MAT	−0.214	0.081	7.026	1	0.008	0.808
	IARP MAT	0.092	0.029	10.132	1	0.001	1.096
	Constant	1.156	1.497	0.596	1	0.440	3.176
Step 4	Heart Rate MAT	−0.084	0.037	5.246	1	0.022	0.919
	SI MAT	−0.032	0.011	8.266	1	0.004	0.969
	Respiratory Rate MAT	−0.203	0.087	5.413	1	0.020	0.816
	IARP MAT	0.151	0.042	13.113	1	0.000	1.163
	Constant	5.824	2.657	4.805	1	0.028	338.313

Notes: MAT—mental arithmetic test; IARP—indicator of the adequacy of regulatory processes; SI—Baevsky’s strain index.

**Table 6:** Association of indicators during mental arithmetic test with components of personality type D (multiple linear regression analysis).

Model		Unstandardized Coefficients		Standardized Coefficients		
		B	Std. Error	Beta	t	Sig.
Indicator of regulatory process adequacy						
	(Constant)	52.740	5.728		9.207	0.000
	Type D	−1.574	7.173	−0.041	−0.219	0.827
	Zscore NA	0.073	0.535	0.022	0.136	0.892
	Zscore SI	7.081	2.786	0.370	2.541	0.013
	ZNA × ZSI	0.976	1.907	0.057	0.512	0.610
Respiratory rate						
	(Constant)	18.611	1.366		13.629	0.000
	Type D	−2.860	1.710	−0.321	−1.673	0.099
	Zscore NA	0.036	0.128	0.047	0.285	0.776
	Zscore SI	0.200	0.664	0.045	0.301	0.764
	ZNA × ZSI	0.428	0.455	0.107	0.941	0.350
Baevsky’s strain index						
	(Constant)	77.524	14.763		5.251	0.000
	Type D	−18.840	18.487	−0.198	−1.019	0.311
	Zscore NA	0.346	1.380	0.042	0.251	0.802
	Zscore SI	15.140	7.181	0.318	2.108	0.038
	ZNA × ZSI	2.301	4.914	0.054	0.468	0.641
Heart rate						
	(Constant)	89.311	4.056		22.021	0.000
	Type D	−3.991	5.079	−0.155	−0.786	0.435
	Zscore NA	0.222	0.379	0.100	0.586	0.559
	Zscore SI	2.475	1.973	0.192	1.255	0.214
	ZNA × ZSI	0.908	1.350	0.079	0.672	0.503

Notes: NA—negative affectivity; SI—social inhibition.

## Data Availability

Data regarding this manuscript are available in the Federal State Budgetary Scientific Institution “Research Institute for Complex Issues of Cardiovascular Disease”, Kemerovo, Russia.

## Supplementary Materials

The following supporting information can be downloaded at https://www.mdpi.com/article/10.3390/bs15070852/s1: Supplementary Table S1. Psychophysiological indices of mental stress associated with personality type D (binary logistic regression analysis, forward likelihood ratio): Omnibus Tests of Model Coefficients; Supplementary Table S2. Psychophysiological indices of mental stress associated with personality type D (binary logistic regression analysis, forward likelihood ratio): Model Summary; Supplementary Table S3. Psychophysiological indices of mental stress associated with personality type D (binary logistic regression analysis, forward likelihood ratio): Classification Table.
