# Supplementary material for: Personality Type D and Psychophysiological Stress Reactivity During Mental Stress in Young Healthy Individuals
Source: Behav Sci (Basel). 2025 Jun 24;15(7):852. doi: 10.3390/bs15070852 (PMC12292575; doi:10.3390/bs15070852)
Supplement: Supplementary file 1 [file behavsci-15-00852-s001.zip › behavsci-3625714-supplementary.pdf]

Supplementary Table S1. Psychophysiological indices of mental stress associated with personality type D (binary logistic regression analysis, forward likelihood ratio):

Omnibus Tests of Model Coefficients

		Chi-square	df	Sig.
Step 1	Step	5.652	1	0.017
	Block	5.652	1	0.017
	Model	5.652	1	0.017
Step 2	Step	6.058	1	0.014
	Block	11.710	2	0.003
	Model	11.710	2	0.003
Step 3	Step	7.991	1	0.005
	Block	19.702	3	0.000
	Model	19.702	3	0.000
Step 4	Step	6.104	1	0.013
	Block	25.806	4	0.000
	Model	25.806	4	0.000

Supplementary Table S2. Psychophysiological indices of mental stress associated with personality type D (binary logistic regression analysis, forward likelihood ratio):

Model Summary

Step	-2 Log likelihood	Cox & Snell R Square	Nagelkerke R Square
1	99.232	0.072	0.096
2	93.174	0.143	0.191
3	85.182	0.228	0.305
4	79.078	0.288	0.385

Supplementary Table S3. Psychophysiological indices of mental stress associated with personality type D (binary logistic regression analysis, forward likelihood ratio):

Classification Table

Observed			Predicted		
			Type_D		
			0	1	Percentage Correct
Step 1	Type D	0	19	16	54.3
		1	12	29	70.7
		Overall Percentage			63.2
Step 2	Type D	0	24	11	68.6
		1	11	30	73.2
		Overall Percentage			71.1
Step 3	Type D	0	28	7	80.0
		1	11	30	73.2
		Overall Percentage			76.3
Step 4	Type D	0	27	8	77.1
		1	11	30	73.2
		Overall Percentage			75.0
